# Inhibitory and excitatory responses in the dorso-medial prefrontal cortex during threat processing

**DOI:** 10.3389/fnins.2022.1065469

**Published:** 2023-01-09

**Authors:** Venkata C. Chirumamilla, Gabriel Gonzalez-Escamilla, Benjamin Meyer, Abdul Rauf Anwar, Hao Ding, Angela Radetz, Tamara Bonertz, Sergiu Groppa, Muthuraman Muthuraman

**Affiliations:** ^1^Section of Movement Disorders and Neurostimulation, Department of Neurology, Focus Program Translational Neuroscience (FTN), University Medical Center of the Johannes Gutenberg University Mainz, Mainz, Germany; ^2^Neuroimaging Center Mainz, Focus Program Translational Neuroscience (FTN), University Medical Center of the Johannes Gutenberg University Mainz, Mainz, Germany

**Keywords:** cortical excitability, threat processing, paired-pulse TMS, effective connectivity, instructed fear paradigm

## Abstract

**Objective:**

To evaluate cortical excitability during instructed threat processing.

**Methods:**

Single and paired transcranial magnetic stimulation (TMS) pulses were applied to the right dorsomedial prefrontal cortex (dmPFC) during high-density electroencephalography (EEG) recording in young healthy participants (*n* = 17) performing an instructed threat paradigm in which one of two conditioned stimuli (CS+ but not CS-) was paired with an electric shock (unconditioned stimulus [US]). We assessed TMS-induced EEG responses with spectral power (both at electrode and source level) and information flow (effective connectivity) using Time-resolved Partial Directed Coherence (TPDC). Support vector regression (SVR) was used to predict behavioral fear ratings for CS+ based on TMS impact on excitability.

**Results:**

During intracortical facilitation (ICF), frontal lobe theta power was enhanced for CS+ compared to single pulse TMS for the time window 0–0.5 s after TMS pulse onset (*t*(16) = 3.9, *p* < 0.05). At source level, ICF led to an increase and short intracortical inhibition (SICI) to a decrease of theta power in the bilateral dmPFC, relative to single pulse TMS during 0–0.5 s. Compared to single pulse TMS, ICF increased information flows, whereas SICI reduced the information flows in theta band between dmPFC, amygdala, and hippocampus (all at *p* < 0.05). The magnitude of information flows between dmPFC to amygdala and dmPFC to hippocampus during ICF (0–0.5 s), predicted individual behavioral fear ratings (CS+; coefficient above 0.75).

**Conclusion:**

Distinct excitatory and inhibitory mechanisms take place in the dmPFC. These findings may facilitate future research attempting to investigate inhibitory/facilitatory mechanisms alterations in psychiatric disorders and their behavioral correlates.

## Introduction

Transcranial magnetic stimulation (TMS) is a brain stimulation technique that offers the opportunity to non-invasively evaluate cortical excitability and interregional connectivity in humans (McMackin et al., 2019b; Belardinelli et al., 2021). Paired pulse TMS allows the investigation of circuits of inhibition and facilitation by applying a subthreshold conditioning stimulus followed by a suprathreshold test stimulus (Ferreri et al., 2011). With interstimulus intervals (ISIs) of 1–5 ms, the responses are inhibited, whereas with longer ISIs of 8–15 ms the responses are facilitated, responses that are referred to as short intracortical inhibition (SICI) and intracortical facilitation (ICF), respectively. However, the mechanisms undelaying these circuits of inhibition or facilitation remain incompletely understood. In this regard, electroencephalography (EEG) can be used to record direct responses to TMS in a given scalp region with the millisecond resolution (Gonzalez-Escamilla et al., 2018; Chirumamilla et al., 2019; McMackin et al., 2019a). TMS-EEG paradigms have been employed to investigate how TMS modulates the intrinsic oscillatory activity and effective connectivity of local and distributed networks (Hordacre et al., 2019).

Among cortical regions, the dorsomedial prefrontal cortex (dmPFC) is involved in situations that require immediate attention and goal-directed behavior. The dmFC is part of the default mode network and has a central role in the generation and regulation of the emotional state (Lindquist et al., 2012; Gonzalez-Escamilla et al., 2018). One common and effective way to investigate emotion regulation are instructed threat paradigms (Gonzalez-Escamilla et al., 2018). In these paradigms, the participants are explicitly informed that a conditioned stimulus (CS+) will be repeatedly paired with an aversive unconditioned stimulus (US), whereas the second stimulus is unpaired (CS-). The subjective fear ratings indicate fear for the stimuli in participants throughout paradigm (Meyer et al., 2015).

Previous research indicates that the awareness about the CS+/US contingency evokes neural activity responses of a network of brain regions, composed by the dmPFC, the amygdala, and hippocampus, among whom the dmPFC dynamically regulates cortical excitability (Phelps et al., 2001; Mechias et al., 2010). The prefrontal-amygdala oscillatory synchrony in the theta frequency (4–8 Hz) contributes to fear expression in rodents (Karalis et al., 2016). Converging with this animal study, recent human studies showed increased theta oscillations at frontal EEG electrodes during CS+ compared to CS- (Chien et al., 2017) and can be modulated by single pulse TMS over the right dmPFC (Chirumamilla et al., 2019). These studies indicate that theta oscillations are crucial in fear processing and in linking brain areas within functional networks. However, it remains unclear whether and how the balance between inhibition and excitation in the right dmPFC regulates behavioral responses during fear processing in humans.

In a previous study, we analysed the transient modulated oscillatory activity measured with EEG and TMS over the right dmPFC during an instructed threat paradigm (Chirumamilla et al., 2019). We showed that single pulse TMS over the dmPFC led to increased theta power at specific time windows during CS+ relative to CS-. Based on these results, in the current study we first focused on characterizing the TMS-EEG neural signature of SICI and ICF in the right dmPFC in terms of spectral modulation at the electrode-level and source space. Subsequently, we explored the effect of SICI and ICF on information flows among dmPFC, amygdala, and hippocampus during the instructed threat paradigm. Finally, to investigate whether information flows alterations are related to fear while viewing CS+, we quantified the relationship of behavioral fear ratings for CS+ and information flows among dmPFC, amygdala, and hippocampus. It was hypothesized that: (Belardinelli et al., 2021) ICF would increase frontal theta activity and information flows among studied brain regions compared to single pulse TMS; (McMackin et al., 2019b) SICI would result in decreased frontal theta power and information flows among the studied three brain regions compared to single pule TMS.

## Materials and methods

### Subjects

Seventeen young healthy subjects (9 males, mean age 28.1±3.5 years) participated in this study. The local ethics committee in the Medical Faculty of the Johannes Gutenberg University in Mainz approved the study protocol, and all participants provided informed written consent at the beginning of the experiment.

### MRI data acquisition

MR images were acquired for the purpose of neuronavigation using a 3-Tesla magnetic resonance imaging (MRI) scanner (Magnetom Tim Trio, Siemens Healthcare, Erlangen, Germany) equipped with a 32-channel head coil at the Neuroimaging Center (NIC) in Mainz, Germany. A T1-weghted magnetization- prepared rapid gradient-echo (MP-RAGE) sequence (repetition time = 1,900 ms; echo time = 2.54 ms; inversion time = 900 ms; pixel bandwidth = 180; acquisition matrix = 320 × 320; flip angle = 9°; pixel spacing = 0.8125; slice thickness = 0.8 mm) was used.

## Experimental procedures

### Instructed threat paradigm

**FIGURE 1:**
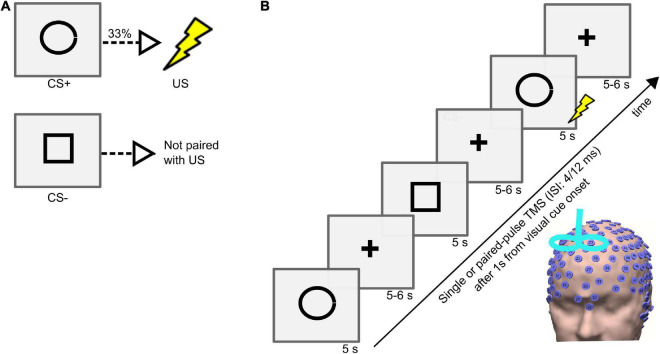
**(A)** Conditioned stimulus (CS+), unconditioned stimulus (US), and neutral stimulus (CS–) and their contingencies in the instructed threat paradigm. **(B)** Trial sequence in the instructed threat paradigm. Each trial consisted of presenting a visual cue (CS+ or CS-) followed by a fixation cross. The visual cue was presented on a computer screen for 5 s followed by a fixation cross that jittered between 5 and 6 s. Single/paired-pulse (with an interstimulus interval (ISI) of 4 or 12 ms in different sessions) TMS was delivered in every trial, 1 s after every visual cue onset.

The instructed threat paradigm was programmed in Matlab (2006b, MathWorks) with the Cogent toolbox.^1^ During the experiment, the participants were seated comfortably on a chair, and an electric shock was applied to the dorsal part of the left hand through a surface electrode that was connected to a DS7A electrical stimulator (Digitimer). The subjects rated the perceived pain on a scale from 0 (no pain) to 10 (intense pain). The electric shock intensity analogous to a pain level of 7 was applied during the experiment. The experiment comprised two visual cues, specifically a circle and a square, that were presented in a pseudo-random order on a computer screen for 5 s with an inter-trial interval (ITI) varied between 9 and 11 s (Figure 1). Before starting the experiment, all the subjects were instructed that a CS+ (visual cue circle) would be paired with a US (electric shock) with a probability of 33% during the time the visual cue was present on the screen, and that the CS- (visual cue square) would never be associated with a shock. In the experiment, the visual cues were counterbalanced across subjects, with half receiving a circle and half receiving a square as visual cue for the CS+. There were four different sessions separated by 5-minute breaks during the experiment. In each session, there were 68 visual cues (41 CS+ including 14 CS+/US, 27 CS-). The stimulation type varied on a trial-by-trial basis. During the experiment, the EEG signals were recorded using a high-density (256 electrodes) EEG system (Net Station 5.0, EGI, United States of America). The electrode impedances were kept below 50 KΩ throughout the experiment (Ferree et al., 2001).

### Transcranial magnetic stimulation

The single and paired pulse TMS of the right dmPFC were applied via two Magstim 200 stimulators (Magstim, United Kingdom) connected through a Bistim unit and delivered through a 70 mm figure-of-eight coil. The resting motor threshold (RMT) was determined as the minimum stimulator output (MSO) required to elicit motor evoked potentials (MEPs) of amplitude 50 μV in 5 out of 10 consecutive trials at rest (Groppa et al., 2012). The single pulse TMS was delivered at 110% of the RMT, while in the paired pulse TMS, a subthreshold conditioning pulse (90% of RMT) was followed by a supra-threshold test pulse (110% RMT) (Garry and Thomson, 2009). The ISIs between the conditioning pulse and test pulse were 4 ms and 12 ms to test SICI and ICF, respectively (Maeda et al., 2002; Heide et al., 2006). The TMS pulses were applied at the right dmPFC (MNI coordinate: *x* = 10, *y* = 12, *z* = 58) (Chirumamilla et al., 2019; Meyer et al., 2019). We used a TMS-Navigator (Localite, Sankt Augustin, Germany) assisted by the individual T1-weighted image to navigate the TMS coil and to maintain its exact location and orientation throughout an experimental session. During the experiment, the subjects wore earplugs that attenuated the clicking sound associated with the TMS pulse.

### EEG data analysis

The EEG data were processed using MATLAB R2015B (Mathworks, USA) with in-house customized analysis scripts and the open-source MATLAB toolbox Fieldtrip (Oostenveld et al., 2011). First, the continuous EEG data acquired during the instructed threat paradigm were divided into epochs from −2 to 5 s relative to TMS pulse onset. Afterward, the EEG data that contained the TMS pulse ringing artifacts (from 0.005 s prior to and 0.02 s after the TMS pulse) were removed. In all the participants, the trials in which electric shock was administered were excluded from further analysis. Then, the resulting EEG data were re-referenced to a grand average of all electrodes. Afterward, the data were visually inspected, and noisy trials were discarded. In addition, the independent component analysis (ICA) was implemented, and the components related to the eye-blinks, muscle and decay artifacts were removed (Hyvärinen, 1999). Finally, the remaining ICA components were transformed back into electrode data representation.

### Behavioral fear ratings

At the end of each paradigm session, all the participants rated their perceived level of fear during the experiment, for both CS+ and CS- independently, on a scale from 0% (not fearful/safe) to 100% (very fearful).

### Time-frequency analysis

The time-frequency analysis technique was implemented on the EEG data to characterize TMS-induced oscillations with a multitaper method (Herring et al., 2015). We applied a Hanning taper to an adaptive time window of 3 cycles for each frequency. The time-frequency grand averages were computed for both single and paired pulse TMS paradigms in the time range of 0–0.5 s (relative to TMS pulse onset) and frequencies ranging from 4 to 7 Hz.

### Source and connectivity analysis

Dynamic imaging of coherent sources (DICS) beamforming was applied to the EEG data to estimate the power of the oscillatory activity originating from the cortex (Gross et al., 2001). The DICS algorithm uses the cross-spectral density (CSD) matrix and forward modeling of the neural currents (Herring et al., 2015). The CSD matrix was determined from 0.5 s of EEG data following single and paired pulse TMS by means of Fast Fourier Transform (FFT) in the theta frequency band. The lead-field matrix (LFM) was modeled using the standard T1-weighted magnetic resonance template image taken from Fieldtrip with the boundary element method (BEM) with three layers (brain, skull, and skin) (Muthuraman et al., 2019). A 3-D grid with 1 cm^3^ resolution was implemented in this study. The spatial filter was calculated using the CSD matrix and the lead field matrix. The estimated spatial filter was applied to the Fourier transformed data, and power was calculated at the source space for the single and paired pulse TMS paradigms separately. Based on our previous findings (Gonzalez-Escamilla et al., 2018), we extracted the pooled source signals from three brain regions, namely dmPFC, amygdala, and hippocampus of both brain hemispheres following the automated anatomical labeling atlas (Tzourio-Mazoyer et al., 2002). Finally, the connectivity values among brain regions (time range: −1.5 to 1.5 s) were estimated using the temporal partial directed coherence method (TPDC). The TPDC method has been described previously (Muthuraman et al., 2018). The TPDC is a time-frequency causality technique, which is used to infer the strength of time-varying causal information flow among brain regions in a specific frequency (Anwar et al., 2013). It has been shown that the multivariate non-stationary time series *x*(*t*) can be modeled using adaptive autoregressive models as shown in Eq. 1 (Arnold et al., 1998).


(1) $$x\,\left(t\right)=\sum_{r=1}^{p}A_{r}\,\left(t\right)\,x\,\left(t-r\right)+e\,\left(t\right)$$

Where *A*_*r*_(*t*) represents the time-varying multivariate autoregressive (MVAR) coefficients, *p* is the model order that can be determined by Akaike Information Criteria (AIC), and *e*(*t*) denotes the zero-mean Gaussian noise. The time-varying MVAR coefficients are estimated by Dual Extended Kalman Filter (DEKF) based on state-space models that are defined by two equations: a state equation, Eq. 2 that connects the previous state with present state and a measurement equation, Eq. 3 which relates state with the observation.


(2) $$x_{t+1}=F\,\left(x_{t},w\right)+n\,\left(t\right)$$

(3) $$y_{t}=C\,x_{t}+\eta \,\left(t\right)$$

Where *n*(*t*) and η(*t*) are white, zero-mean Gaussian noise processes, *w* denotes model parameters, *x*_*t*_ is the state at the time *t*, and *y*_*t*_ is the target time series. In this approach, both states and model parameters of the system are estimated recursively at each time point. At each time point, these parameters are Fourier transformed, and partial directed coherence (PDC) is calculated. The PDC is a connectivity measure that can differentiate the direct and indirect connections in a particular frequency. The PDC from the region _‘_ to region *i* is calculated as per Eq. 4 (Ghumare et al., 2018).


(4) $${\text{PDC}}_{i,j}\,\left(f\right)=\frac{\left|A_{i\,j}\,\left(f\right)\right|}{\sqrt{k\,{\left|A_{k\,j}\,\left(f\right)\right|}^{2}}}$$

Where *A* denotes the Fourier transformed MVAR coefficients. After calculating PDC at each time point, all the values are concatenated, yielding the TPDC (Pester et al., 2015).

### Statistical analysis

The statistical analyses were performed with MATLAB (R2015b, The Mathworks^®^) and SPSS 23.0 (IBM, Armonk, NY, USA). The post hoc tests were performed with the Bonferroni correction method. The *p*-value of < 0.05 was considered to be statistically significant. The significant difference between the stimulus conditions (CS+ and CS-) in behavioral fear ratings were tested with the paired *t*-test. To study the effect of TMS stimulation paradigms on theta power in the frontal lobe during CS+, we performed a one-way analysis of variance (ANOVA) with factor stimulation (3 levels: single pulse, ICF, and SICI). To investigate the effect of stimulation paradigms on connectivity, we carried out three-way repeated measures ANOVA (rmANOVA) with factors time (6 levels: −1.5 to −1, −1 to −0.5, −0.5 to 0, 0 to 0.5, 0.5 to 1, and 1 to 1.5 s), direction (6 levels: dmPFC to amygdala, dmPFC to hippocampus, hippocampus to amygdala, hippocampus to dmPFC, amygdala to dmPFC, and amygdala to hippocampus) and stimulation (3 levels: single pulse, ICF, and SICI).

Finally, we performed a support vector regression (SVR) to predict the individual fear ratings (CS+ and CS-) using the information flows values among derived from the connectivity of the a priori selected brain regions dmPFC, amygdala, and hippocampus during different stimulation conditions. Here, outcome regression coefficients of 0.7 obtained after 5-fold cross-validation were considered as a large effect.

## Results

### Behavioral data

**FIGURE 2:**
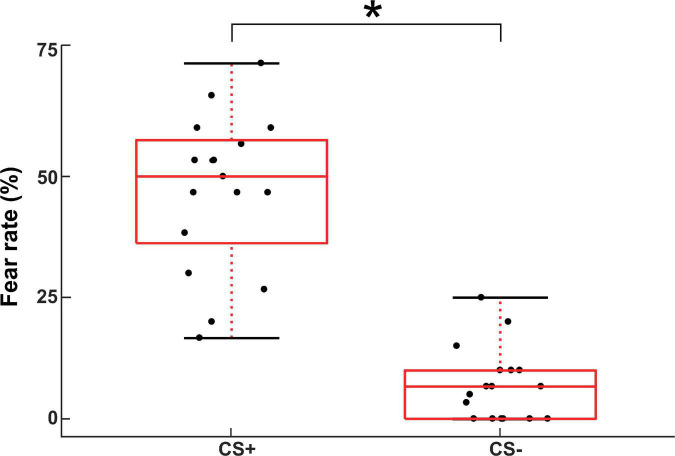
Subjective fear ratings for the CS+ and CS- conditions in the instructed threat paradigm. The asterisk (*) denotes significant difference (*p* < 0.001).

The measured RMT values across subjects were 59.7 ± 5.9 MSO. The mean values of subjective fear ratings for the CS+ and CS- conditions in the instructed threat paradigm were 46.2 ± 21 and 13.2 ± 17.1, respectively. The fear ratings indicated a well induced fear in the participants for the CS+ relative to the CS- condition in the paradigm (*t*(16) = 11.3; *p* < 0.001; Figure 2).

### ICF enhances theta power

**FIGURE 3:**
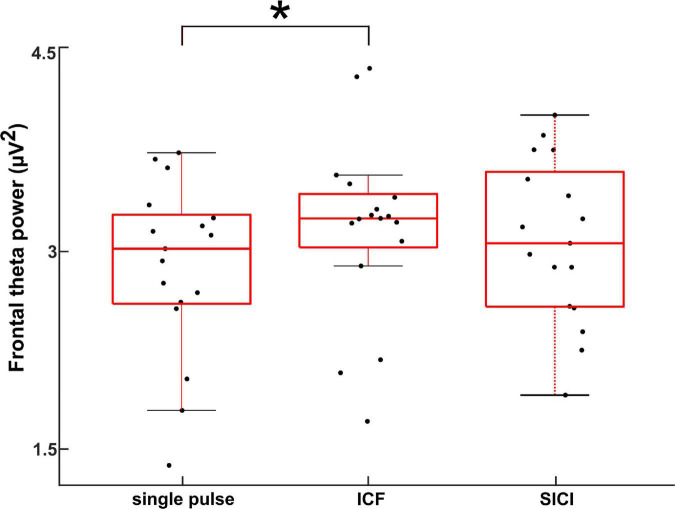
Electroencephalography power during CS+ trials in the sensor space obtained by averaging across frontal electrodes (Chirumamilla et al., 2019) in the theta band for different stimulation paradigms (single pulse, ICF, and SICI) for the time range of 0–0.5 s (relative to TMS pulse). The asterisk (*) denotes significant difference (*p* < 0.001).

**FIGURE 4:**
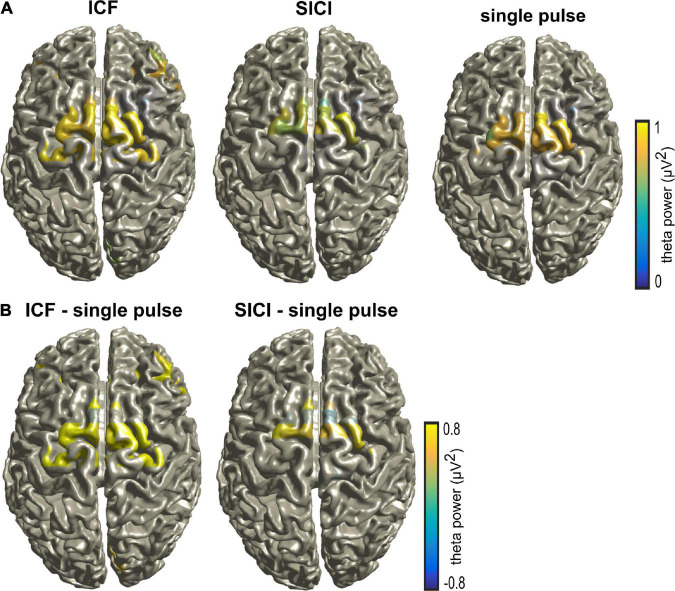
**(A)** Source activity during CS+ trials in the theta band for different stimulation paradigms (single pulse, ICF, and SICI) for the time range of 0–0.5 s (relative to TMS pulse). **(B)** The differences between different stimulation paradigms ICF – single pulse and SICI – single pulse, respectively. Note that the source activity was thresholded to show the maximal activity.

Figure 3 shows the theta power during CS+ trials across the different stimulation paradigms. Theta power was calculated between 0 and 0.5 s (relative to TMS pulse) and averaged across all the subjects and the frontal lobe electrodes (shown in Supplementary Figure 1). The ANOVA on frontal lobe theta power during CS+ revealed a significant main effect for the factor stimulation (*F*(2,48) = 81.249, *p* < 0.005). The post hoc comparisons showed that the frontal lobe theta power during CS+ was larger during ICF compared to single pulse (*t*(16) = 3.9, *p* < 0.005; Figure 3). In contrast, there were no differences in theta power during CS- across different stimulation paradigms (*p* > 0.05). The two-way ANOVA on frontal lobe theta power showed a significant main effect for the factor stimulation and significant two-way interaction of stimulation and stimulus as shown in Supplementary Table 1. The post hoc comparisons showed that the frontal lobe theta power during CS+ was larger during ICF compared to a single pulse (*t*(16) = 2.34, *p* < 0.05). We found maximal source activity in the bilateral dmPFC for the different stimulation paradigms in the theta band for CS+ trials in the time of 0–0.5 s (Figure 4). Particularly, we observed increased activity during ICF while decreased during SICI relative to single pulse with the peak activity in the right dmPFC.

### ICF and SICI alter the information flows

**FIGURE 5:**
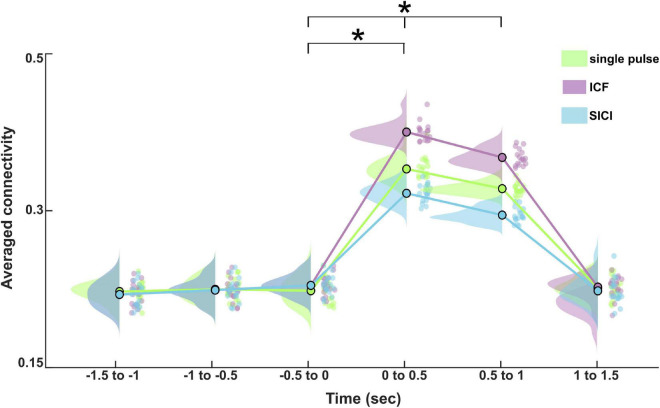
Average effective connectivity dynamics in the studied network for different stimulation paradigms (single pulse, ICF, and SICI) during CS+ in the theta band at the source level. The asterisk (*) denotes the significant difference after correcting for multiple comparisons (*p* < 0.001, Bonferroni corrected).

**FIGURE 6:**
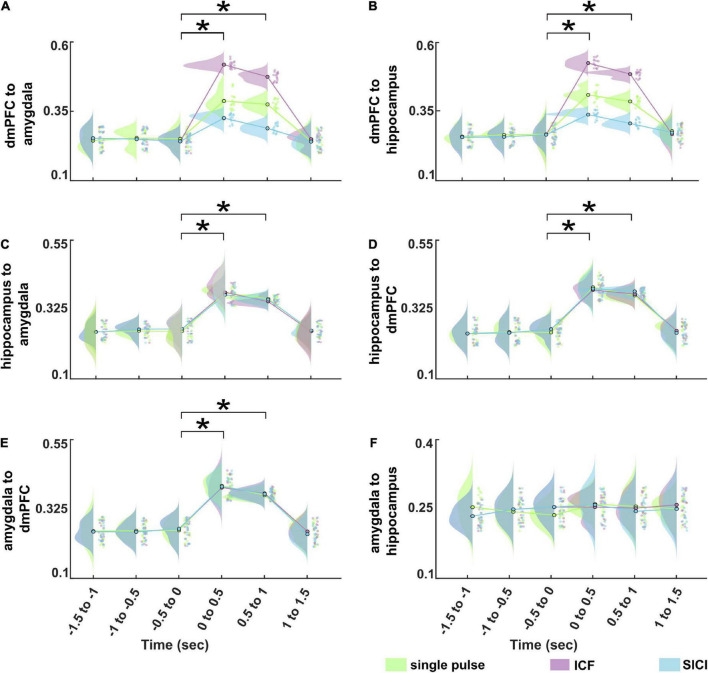
Information flows dynamics for different stimulation paradigms (single pulse, ICF, and SICI) during CS+ in the theta band at the source level between **(A)** dmPFC to amygdala,**(B)** dmPFC to hippocampus, **(C)** hippocampus to amygdala, **(D)** hippocampus to dmPFC, **(E)** amygdala to dmPFC, **(F)** amygdala to hippocampus, respectively. The asterisk (*) denotes the significant difference after correcting for multiple comparisons (*p* < 0.001, Bonferroni corrected).

We determined the information flows (connectivity values from dmPFC to amygdala, dmPFC to hippocampus, hippocampus to amygdala, hippocampus to dmPFC, amygdala to dmPFC, and amygdala to hippocampus) at the source level as measured by TPDC from −1.5 to 1.5 s (relative to TMS pulse onset) divided into six non-overlapping time windows. Figure 5 shows the average TPDC across connections for different stimulation paradigms during CS+ in the analyzed time windows in the theta band. The three-way rmANOVA showed a statistically significant three-way interaction of stimulation, time and direction as seen in Table 1. We also found significant two-way interactions: stimulation*time, stimulation*direction and time*direction and significant main effects for stimulation, time, and direction. Bonferroni-corrected post hoc tests for the factor time revealed a significant increase in the connectivity in the time windows 0–0.5 s and 0.5–1 s compared to baseline time window (−0.5 to 0 s) (all *p*’s < 0.001). Furthermore, the post hoc tests on the factor direction indicated higher connectivity between dmPFC to hippocampus compared to hippocampus to dmPFC (*p* < 0.001). The post hoc tests on the factor stimulation showed that the connectivity was significantly increased during ICF, whereas it was reduced during SICI relative to the single pulse (both *p*’s < 0.001). This difference between stimulation paradigms was evident from the connections dmPFC to hippocampus and dmPFC to amygdala showing higher connectivity in ICF and lower connectivity in SICI compared to single pulse in the time windows (0–1 s) (all *p*’s < 0.01; Figure 6). In summary, paired pulse TMS on the right dmPFC during CS+ in an instructed threat paradigm led to alterations of information flows among dmPFC, hippocampus and amygdala.

**TABLE 1:** The rmANOVA with dependent variable of TPDC, and stimulation, time, and direction as independent variables.

	Sum of squares	(df intercept, df error)	*F*	*p*
Stimulation	0.14	(2, 1,728)	98.3	<0.005
Time	5.85	(5, 1,728)	1641.7	<0.005
Direction	0.60	(5, 1,728)	169.8	<0.005
Stimulation*Time	0.26	(10, 1,728)	37.7	<0.005
Stimulation*Direction	0.24	(10, 1,728)	36.3	<0.005
Time*Direction	1.05	(25, 1,728)	59.2	<0.005
Stimulation*Time*Direction	0.52	(44, 1,584)	17.1	<0.005

*Means interaction between factors.

### Relationship between information flows and behavioral fear ratings

**FIGURE 7:**
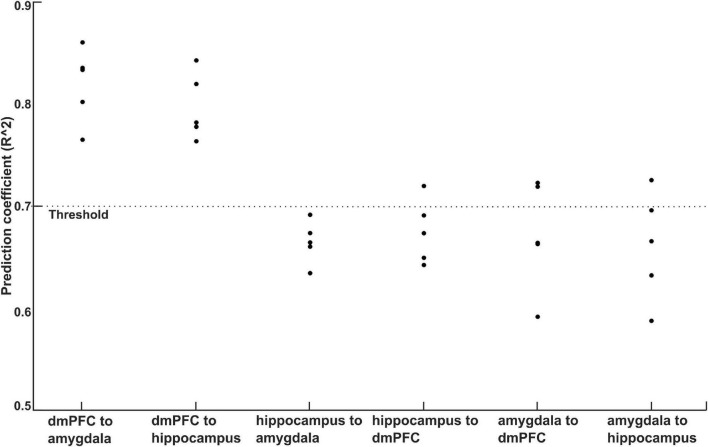
Plot of prediction accuracy obtained for behavioral ratings of CS+ using SVR analysis of information flows among dmPFC, amygdala, and hippocampus. The cross-validated (5-fold) prediction coefficients was above 0.75 for the information flows between dmPFC to amygdala, and dmPFC to hippocampus.

We performed SVR analyses to quantify the relationships between TMS induced changes in information flows (0–0.5 s relative to −0.5 to 0 s) and behavioral fear rating for CS+ and CS-. In this analysis during ICF, we found cross-validated averaged SVR coefficients of 0.81, and 0.79 for information flows between dmPFC to amygdala, and dmPFC to hippocampus, respectively (Figure 7). The SVR analysis on information flows among other brain regions found averaged SVR coefficient of 0.66 for hippocampus to amygdala, 0.67 for hippocampus to dmPFC, 0.67 for amygdala to dmPFC, and 0.66 for amygdala to hippocampus. Taken together, the higher SVR coefficient for information flows between dmPFC to amygdala, and dmpFC to hippocampus among other connections indicates the stimulation effect of dmPFC. During the SICI and single pulse, all the averaged SVR coefficients obtained for the information flows among the studied brain regions were not significant (all coefficients < 0.7).

## Discussion

The main objective of the present study was to investigate cortical excitability by means of single and paired pulse TMS over the right dmPFC, as reflected on human brain oscillatory activity. For this, we investigated information flows within the core nodes of the fear processing network during an instructed threat paradigm. Within our framework of simultaneous TMS and EEG, we demonstrated that the application of specific TMS pulse stimulation paradigm over the dmPFC has a direct impact on the excitatory and inhibitory circuits of the neural oscillatory activity and the information flows within a specific time window. Furthermore, we have identified that the alterations in information flows evoked by ICF predicted the behavioral fear ratings of the conditioned stimulus (CS+). Our findings pave the way for the future assessment of facilitatory and inhibitory physiological activity in the prefrontal cortex for patients with psychiatric and affective disorders.

### Theta power modulation over dmPFC

Previous research using EEG neural oscillations in humans (Meyer et al., 2015; Chien et al., 2017) has proposed that fear processing is associated with increased theta power in frontal electrodes during CS+ relative to CS- trials (Chien et al., 2017). These alterations may likely reflect or be related to theta activity in the amygdala and hippocampus. Accordingly, here we showed that the excitability state of the right dmPFC directly impacted the spectrum of theta oscillations. Specifically, we found that the frontal lobe theta power was enhanced during ICF compared to single pulse but not for SICI during CS+ trials. Further, the EEG source analysis showed that ICF and SICI induced significant alterations of source power in the theta band compared to single pulse. Particularly, theta source power increased during ICF and decreased in SICI compared to single pulse in the bilateral dmPFC. Taken together, these results suggest that theta band oscillations may mediate the communication from dmPFC and other regions.

### Distinct information flows fingerprints in response to ICF and SICI

According to the previous results, we characterized whether the information flows between the dmPFC, amygdala, and hippocampus differed among different excitability paradigms (i.e., single and paired pulse (SICI and ICF) TMS stimulation paradigms) using the TPDC method. We focused on CS+, rather than CS-, as we did not find any significant differences in CS- related oscillatory activity between stimulation paradigms within the theta band. The findings again suggested that the entrainment of theta oscillations led the differences between single pulse TMS, SICI, and ICF on information flow, particularly ICF increased the information flows from dmPFC to the hippocampus and amygdala when compared to single pulse TMS, whereas SICI suppressed the information flows. The ICF findings may explain the previously reported the overall increase in EEG theta power response to threat stimuli after single pulse TMS over the right dmPFC in (Chirumamilla et al., 2019). Our findings are also concordant with studies in mice where the theta oscillations are considered as a mechanism mediating prefrontal-amygdala coupling related to fear expression (Karalis et al., 2016). Furthermore, in mouse, some medial PFC neurons are modulated by hippocampal theta oscillations (Lesting et al., 2011). Overall, the current findings propose that cortical excitability state of the dmPFC has a direct impact on its theta-mediated communication with subcortical nodes.

### Excitability-related information flows dynamics predict behavior

In our study, SVR analysis showed that the behavioral fear responses can be predicted from information flows alterations elicited by ICF but not by SICI. Particularly for the connections from dmPFC to hippocampus and dmPFC to amygdala within the theta band. The higher predictive power of ICF paradigm might be due to the existence of multiple synaptic connections compared to SICI (Iscan et al., 2016). However, further studies addressing the molecular mechanisms of cortical excitability are needed to fully shed light onto this associations.

## Limitations

There are some limitations to the current study. First, the stimulation parameters were defined based on the motor cortex excitability. This is due to the lack of a reliable method for calculating the TMS intensities outside the motor cortex (Pell et al., 2011). Second, we did not mask the noise caused by the TMS click by playing auditory background noise through headphones. However, we aimed at reducing this possible confound by asking the participants to wear ear-plugs during the whole experiment (Chirumamilla et al., 2019). Finally, we did not determine the dmPFC coordinate for each participant based on individual task-fMRI-related activations. However, we did use the dmPFC coordinate based on the previous fMRI study that showed activation of dmPFC during CS+ compared to CS- trials in an instructed threat paradigm (Meyer et al., 2019). Furthermore, we employed neuronavigation to target the right dmPFC with TMS. The realistic sham TMS pulses were not applied to the right dmPDC due to time constraints in the experimental study design. Future studies are needed utilizing this study design and sham stimulation.

## Conclusion

In the present study, we demonstrated the distinct inhibitory and facilitatory effects of paired pulse TMS on neural processing during fear processing. Furthermore, the results show the presence of functional connections among dmPFC, amygdala, and hippocampus during fear processing, and accentuate the utility of TMS in determining the information flows patterns among brain regions. Our results indicate that information flows alterations between dmPFC to amygdala, and dmPFC to hippocampus during ICF are the best predictors of behavioral fear ratings (CS+). Overall, the current results may be extended for the research evaluating the alterations of intracortical inhibition and facilitation in neurological or psychiatric diseases and associate them with particular behavioral outcomes.

## Data availability statement

The original contributions presented in this study are included in this article/Supplementary material, further inquiries can be directed to the corresponding author.

## Ethics statement

The studies involving human participants were reviewed and approved by Ethics Committee in the Medical Faculty of the Johannes Gutenberg University in Mainz. The patients/participants provided their written informed consent to participate in this study.

## Author contributions

VC contributed to the data acquisition, data analysis, and manuscript writing. GG-E, AA, HD, and AR contributed to the data discussion and manuscript writing. TB and BM contributed to the data acquisition. SG and MM contributed to the experimental design, data analysis, and revision of the manuscript. All authors contributed to the article and approved the submitted version.

## Footnotes

1
http://psychtoolbox.org/links

## Supplementary material

Supplementary Figure 1 The EEG electrodes corresponding to the frontal lobe.
