# Supplementary material for: Inhibitory and excitatory responses in the dorso-medial prefrontal cortex during threat processing
Source: Front Neurosci. 2023 Jan 9;16:1065469. doi: 10.3389/fnins.2022.1065469 (PMC9868831; doi:10.3389/fnins.2022.1065469)
Supplement: Supplementary file 1 [file Table_1.docx]

Supplementary Table 1. The two-way ANOVA with dependent variable of frontal theta power, and stimulation and stimulus as independent variables.

|  | Sum of squares | (df intercept, df error) | F | p |
| --- | --- | --- | --- | --- |
| Stimulation | 118.99 | (2,96) | 4.29 | < 0.05 |
| Stimulus | 36.25 | (1,96) | 2.62 | > 0.05 |
| Stimulation*Stimulus | 112.24 | (2,96) | 4.05 | < 0.05 |
