# Supplementary figures and images for: Inhibitory and excitatory responses in the dorso-medial prefrontal cortex during threat processing
Source: Front Neurosci. 2023 Jan 9;16:1065469. doi: 10.3389/fnins.2022.1065469 (PMC9868831; doi:10.3389/fnins.2022.1065469)

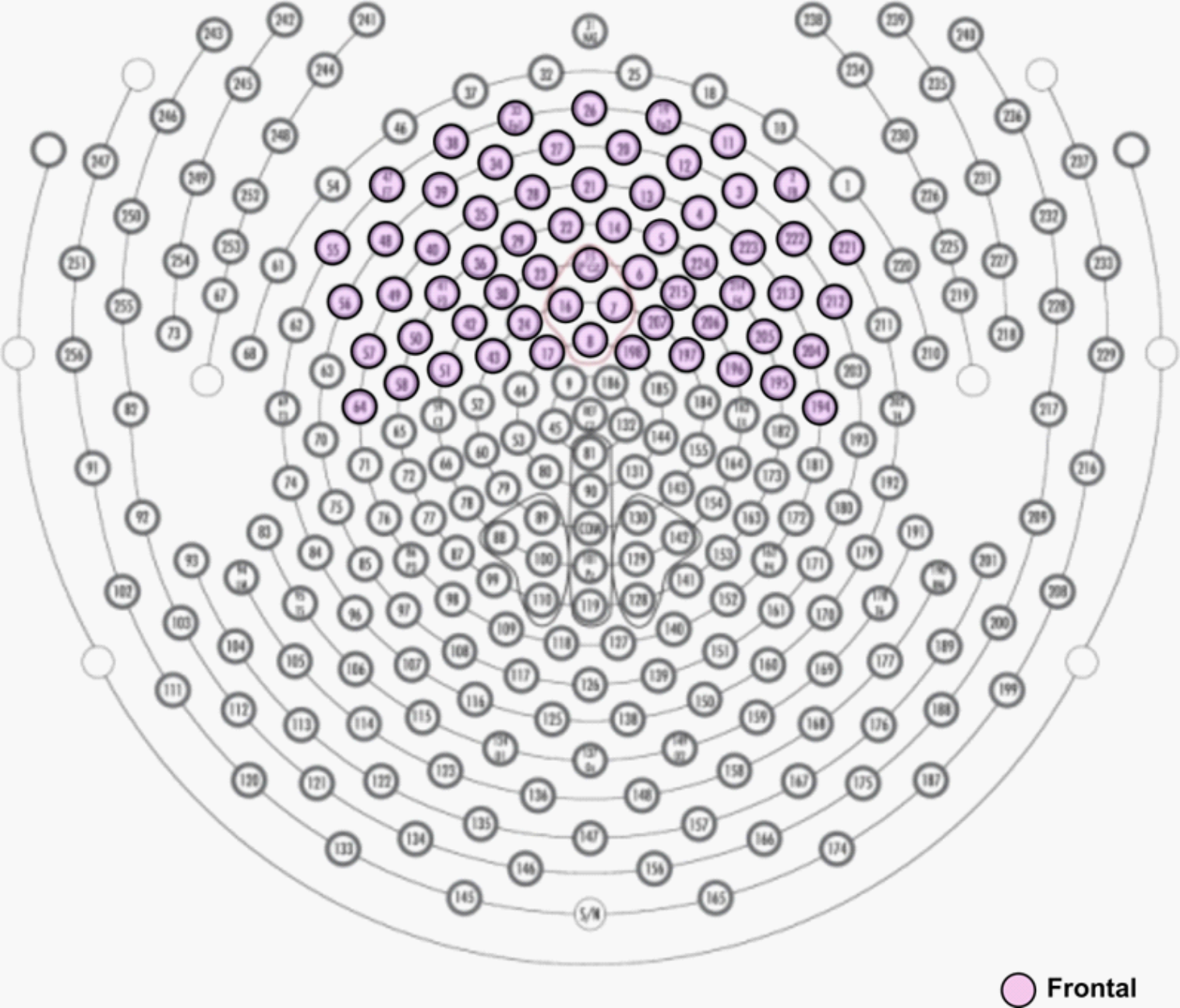

Supplement: Supplementary Figure 1 — The EEG electrodes corresponding to the frontal lobe. [file Image_1.TIF]
